# Encouraging Patient Engagement Behaviors from the Perspective of Functional Quality

**DOI:** 10.3390/ijerph17228613

**Published:** 2020-11-19

**Authors:** Yi Mei, Xiaoyan Xu, Xiaodong Li

**Affiliations:** 1 School of Management, Zhejiang University, Hangzhou 310058, China; 11620034@zju.edu.cn; 2 School of Economics and Management, Anhui Polytechnic University, Wuhu 241005, China

**Keywords:** functional quality, patient engagement behaviors, trust, satisfaction, high-contact professional services

## Abstract

Despite extensive research on how patient engagement behaviors (PEBs) are facilitated though explicit technical interventions in medical services, research on the encouragement of PEBs from the perspective of the service process is lacking. This study explores how functional quality dimensions (responsiveness, empathy, surroundings, and access) affect PEBs (compliance and loyalty) through a two-channel psychological mechanism (trust and satisfaction). This study tests the proposed model using survey data from two public hospitals in southeastern China and employs the partial least square (PLS) technique of structural equation modeling (SEM) to analyze the data. The results show that service providers’ responsiveness, empathy, and access affect patient compliance and loyalty through patient satisfaction; however, the effect of surroundings is not significant. The responsiveness and empathy of service staff affect PEBs through patient trust. Considering the high-contact professional nature of medical services, we call for more efforts toward improving service processes rather than simply relying on technical interventions. Specifically, hospitals and contact employees should devote time and effort to functional quality management in three dimensions, namely responsiveness, empathy, and access, to secure patient trust and satisfaction.

## 1. Introduction

### 1.1. Background

Medical systems all over the world are faced with the challenges of reducing risks and improving patient health outcomes. Encouraging patient engagement behaviors (PEBs) is widely seen as a way to address these challenges [1]. Patients who are encouraged to engage in complying with doctors’ instructions and adhering to treatment regimens, acquire better treatment results [1]. They are also encouraged to spread positive word-of-mouth (WOM) and engage in return patronage, which contributes to the long-term profitability of medical service providers [2]. Therefore, medical service practitioners strive to determine how to facilitate PEBs. 

Prior research has revealed the importance of PEBs and has provided valuable insights into how PEBs are facilitated through explicit technical interventions [1]. For example, Schrader et al. [3] studied the effect of an online-management program enabling patient–clinician communication on PEBs. Quinn et al. [4] emphasized the need for mobile diabetes interventions to encourage PEBs. However, such technical interventions are costly but not definitively effective and only work for a small number of specific groups who have particular diseases that require long-term treatment and self-service such as depressed, HIV-positive, and chronic patients [1,5,6]. Additionally, the framework proposed in the healthcare intervention literature has not provided a clear psychological mechanism from interventions to PEBs. Third, the previous health research has not paid attention to the essential characteristics of medical services. Medical services are a type of high-contact professional service, characterized by high levels of communication time, intimacy of communication, richness of information exchanged, and a large knowledge gap between providers and patients [7,8]. Patient psychological processes and consequent behaviors are easily influenced by functional aspects across the service process [9]. Therefore, it is necessary to propose a model to describe the formation process of PEBs based on patient experience and the feelings derived from the high-contact professional process of medical service delivery.

### 1.2. Aim

This study aims to fill the abovementioned knowledge gaps by developing a new model to study the formation of PEBs stimulated by the service process rather than by particular interventions. Considering the essential characteristics of high-contact professional services, this study explores how functional quality dimensions (responsiveness, empathy, surroundings, and access) affect PEBs (compliance and loyalty) through a two-channel psychological mechanism of trust and satisfaction. 

## 2. Theoretical Framework and Hypothesis Development

### 2.1. Patient Engagement Behaviors (PEBs)

PEBs refer to those behaviors that reflect patients’ connection with service providers, involve patient resource investment, and add value for patients and providers [10,11,12]. Ideally, patients with sufficient skills, knowledge, and motivation are expected to be actively involved in the entire healthcare system, i.e., from making decisions in direct care to participating in organizational projects, management, and policymaking. In practice, however, this level of patient engagement is very difficult to reach. The reason is that patients prefer to rely on physicians to make diagnostic decisions due to their limited medical knowledge [13], and physicians are often less likely to involve patients in diagnostic decisions due to contextual factors such as their overwhelming workload and time constraints [9]. Previous studies suggested that patients are still able to engage in high-contact professional services and add value by engaging in compliance behaviors [12,14,15,16] and loyalty behaviors [12]. As a PEB, patient compliance consists of a series of behaviors such as complying with rules, following the provider’s instructions, and adhering to the plan formulated by the providers [17]. It reflects a guided partnership between patients and doctors and contributes to better treatment effects and positive health outcomes [1]. Another type of PEB, patient loyalty consists of patient return and referrals [18]. Patient loyalty behaviors involve the investment of patient financial resources (return) and social resources (referral), express patients’ desire to maintain a long-term cooperative relationship with medical staff and hospitals, and contribute to hospitals by increasing sales and profits and expanding the customer base [19].

### 2.2. The Formation Process of PEBs

Considering that PEBs are the specific embodiment of customer engagement behaviors in medical services, we develop a model to describe the process of patient engagement with the assistance of the customer engagement frameworks proposed by Bowden [20] and Ranjan and Read [21]. These two models describe a dynamic process of customer engagement in general services, that is, positive experience leads to valuable behavior through psychological mechanisms. Therefore, the process of patient engagement involves PEBs, psychological mechanisms that drive PEBs, and stimuli for psychological mechanisms. Compared to previous patient engagement frameworks that tend to study PEBs from a technical intervention perspective [1], our framework places more emphasis on such a natural (non-interventional) process whereby a patient’s feelings and subsequent behaviors are spontaneously triggered by the delivery process of high-contact professional services.

#### 2.2.1. Patient Engagement Psychological Mechanisms (PEPMs)

Following the above framework, satisfaction and trust serve as PEPMs. As an affective mechanism and according to the affect theory of social exchange [12], patient satisfaction leads to compliance and loyalty as a form of positive feedback. Moreover, the affective mechanism is inadequate because PEBs require patient resource input; however, the value of PEBs, as evidenced by outcomes such as care results requires time to realize. As a cognitive mechanism, patient trust in the characteristics and capability of providers is a prerequisite for patients to comply with healthcare professionals to obtain positive health outcome as expected. Similarly, patients who trust service providers resist other short-term alternatives in favor of long-term benefits, such as better personalized medical advice [22]. In addition, we suggest that patient trust positively affects patient satisfaction. Patient trust helps to reduce patients’ doubts about their physician’s competence and behavior and will reduce and alleviate their inner anxiety, thus making them more inclined to give positive emotional feedback about the care they receive and the providers of that care. Therefore, the following hypotheses are proposed:

**Hypotheses** **1a** **(H1a).** *Patient satisfaction is positively related to patient compliance.*

**Hypotheses** **1b** **(H1b).** *Patient satisfaction is positively related to patient loyalty.*

**Hypotheses** **2a** **(H2a).** *Patient trust is positively related to patient compliance.*

**Hypotheses** **2b** **(H2b).** *Patient trust is positively related to patient loyalty.*

**Hypotheses** **3** **(H3).** *Patient trust is positively related to patient satisfaction.*

#### 2.2.2. Functional Quality

We adopt functional quality as the stimulus of PEPMs across patient experience for several reasons. First, due to insufficient knowledge to judge the technical aspects of care [22,23,24,25], vulnerable patients focus on functional aspects to judge trustworthiness and reduce their uncertainty about the invisible and uncontrollable nature of service results before taking any action [26]. Second, care results, which are the basis upon which patients judge technical quality, cannot be determined immediately [27]. In addition, a high-contact system causes patients to be highly susceptible to touch points such as contact employees, the surroundings, and access to those touch points. Whether patient interaction with those touchpoints is concordant with the patient’s values, needs, and preferences can largely influence patients’ affect (satisfaction) and cognition (trust), which drive their subsequent engagement behaviors.

Accordingly, we propose that the functional quality of medical services consists of empathy, responsiveness, surroundings, and access. Specifically, drawing on SERVQUAL theory, we choose responsiveness and empathy as the interaction quality dimensions of service staff [28]. Due to patients’ inability or unwillingness to participate in medical decisions and contextual factors such as time constraints, physicians often play the roles of knowledge provider and decision maker in the interpersonal interactions between physicians and patients [13]. The literature shows that the empathy and responsiveness of service staff in this interpersonal process have great potential to influence patient satisfaction by causing patients to feel that physicians can provide the necessary expertise and have designed the medical plan based on the patients’ needs and preferences [29,30]. Surroundings are also chosen from SERVQUAL theory as a narrow concept of tangibles, referring to the ambient conditions and physical facilities in the reception area [31,32]. The literature shows the impact of surroundings on patient satisfaction [18,25] since ambient conditions, such as the temperature, air quality, noise, and smell, can affect patients’ senses and the display of the equipment in a facility can determine whether patients can conveniently use the facility. We do not select assurance and reliability from the SERVQUAL model because scholars have often argued that assurance lacks discriminant validity with empathy [33] and reliability refers to the technical adequacy of professionals, which is beyond nonprofessional patients’ ability to perceive. In addition, we include access, which is related to patients’ perceived time expenditures required to initiate service delivery as a new dimension to describe functional quality because high-contact systems depend on time [34], and a high level of effort exerted by patients to gain access to the service will trigger dissatisfaction [35]. Therefore, the following hypotheses are proposed:

**Hypotheses** **4a** **(H4a).** *Service providers’ responsiveness is positively related to patient satisfaction.*

**Hypotheses** **4b** **(H4b).** *Service providers’ empathy is positively related to patient satisfaction.*

**Hypotheses** **4c** **(H4c).** *Surroundings are positively related to patient satisfaction.*

**Hypotheses** **4d** **(H4d).** *Access is positively related to patient satisfaction.*

As two important interaction dimensions [36], the responsiveness and empathy of service providers can affect patient trust by improving the predictability of service providers. Providers’ responsibility is related to the predictability of service availability, which enables patients to quickly obtain necessary information and services from providers. Similarly, providers’ empathy improves the predictability of service usage in terms of service relevance, which is related to the ability of providers to deliver information and services based on patient preferences. Due to the lack of consultation time and the complexity of disease diagnosis, health professionals can hardly teach patients all the knowledge and information needed to solve their diseases [37,38]. However, health professionals can become responsive and empathetic to gain the trust of patients by providing timely information tailored to their needs and preferences. Therefore, the following hypotheses are proposed:

**Hypotheses** **5a** **(H5a).** *Service providers’ responsiveness is positively related to patient trust.*

**Hypotheses** **5b** **(H5b).** *Service providers’ empathy is positively related to patient trust.*

#### 2.2.3. Control Variables

There is still no consensus on how demographic factors affect PEBs. Thus, we selected several demographic variables, including gender, age, education, and occupation as the control variables.

**Figure 1:**
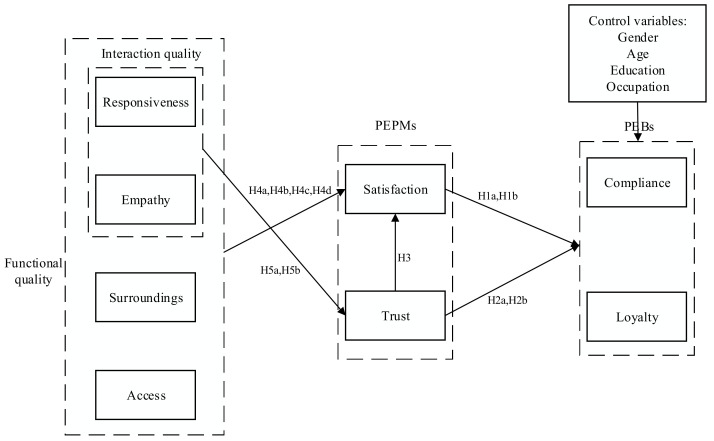
Research model.

Figure 1 illustrates the research model.

## 3. Methods

### 3.1. Setting

The data were mainly collected from a public hospital in southeastern China. We chose this hospital as our target hospital for several reasons. First, our research subjects were patients and the hospital had more than 3 million outpatient visits per year in the last five years. The patients came from all over the country, so we had a sufficient sample for the study and the sample selection was conducive to overcoming regional limitations. Second, the study was rooted in a research context of high-contact professional medical services and our observations and interviews with healthcare professionals and patients revealed that the medical services provided in this hospital were fittingly characterized by high-frequency or prolonged doctor–patient or nurse–patient contact and highly complex diagnoses. Third, 96% of the hospitals in China are public hospitals, and as a comprehensive public hospital with a full range of services and one of the first to be established as a tertiary care hospital, our target hospital was representative. Fourth, this hospital had a good relationship with our project team and greatly facilitated our research, such as by actively participating in the design and revision of the questionnaire and by helping us to administer the questionnaire in intervals, as our scale was spread across two questionnaires. To avoid any adverse effects that the specificity of this hospital might have on the generalizability of the results, additional data were collected at a smaller public hospital fittingly characterized by high-contact professional services. Our study protocol was approved by the ethics committee of the School of Management, Zhejiang University. The protocol number is ZJUSOM20200701.

### 3.2. Participants and Procedures

A separation approach to data collection was adopted to avoid the influences of common method variance. We distributed the questionnaires in two rounds. In the first round, we randomly selected patients on each floor of our target tertiary hospital based on the approximate proportion of the population, and we distributed the questionnaires containing the items on functional quality and psychological mechanisms of patient engagement and questions about demographics and contact information (e-mail address or WeChat). The items on functional quality were responded to in face-to-face interviews, and the rest of the questions were answered by the patients themselves. In the second round, we distributed questionnaires containing items on PEBs by e-mail or by WeChat (China’s most popular social network platform) to the patients who clearly answered all the questions in the first round. The interval between the two rounds was between 6 and 24 h. The interval was long enough to erase patients’ biased memory of the previous stage because they were concerned with their own affairs in such a busy hospital. The practice of collecting independent and dependent variables separately at two points in time to overcome common method variance was also used by Blau [39]. A total of 1000 questionnaires were distributed from April to July 2019. In the first round, 814 valid questionnaires were collected; in the second round, 405 valid questionnaires were collected. Questionnaires with the same answers for all the questions or with many unanswered questions were considered invalid. We repeated the same data collection procedures in this hospital from October to November 2019. In the first round, 800 questionnaires were distributed, and 694 valid responses were obtained, and in the second round, 384 valid responses were obtained. We repeated the same data collection procedures in another smaller hospital from October to November 2020. In the first round, 300 questionnaires were distributed, and 254 valid responses were obtained, and in the second round, 153 valid responses were obtained. Overall, 942 valid questionnaires were collected, for an overall effective response rate of 44.86%. We used Harman’s single-factor test, which has been frequently used in previous research, such as Barbosa et al. [40] and Fang et al. [41], to verify whether there was common method bias. The test showed that there were several different factors, six of which had eigenvalues greater than 1. The amount of variance explained by the first factor was below the threshold of 40%. Therefore, there was no serious common method bias. The demographic characteristics of the samples are shown in Table 1.

The stability of the data for the three periods was assessed using the Wilcoxon matched-pairs signed-ranks test, which is a nonparametric test, since the kurtosis and bias coefficients of all the variables were not equal to zero [18]. The test results revealed no systematic differences in the answers for any of the indicators, indicating that our data were stable across time. We evaluated the nonresponse bias by comparing the demographics of our pooled samples with the archival data of the hospitals on the patient population, and no systematic difference was found. Therefore, this study had no response bias [42].

### 3.3. Measures

The items in the scale were selected from the existing scales used in a large number of empirical studies. We used three phases of content and face validity testing to guarantee the reliability and validity of the selected items. The first phase involved five academic experts in the field of service marketing, who were invited to evaluate the face validity and content validity of each item. The percentage of absolute agreement was used to assess inter-rater agreement between experts [43]. The percentage of absolute agreement on each item ranged from 90% to 100%, meaning that the experts agreed that most of the items performed well in representing their respective constructs. In the second phase, the revised items were reviewed by nine experts from the target tertiary hospital management team, including the dean, directors of various departments, senior doctors and nurses, and the IT staff who were regularly involved in the preparation of the hospital’s own online questionnaire for patients. Those experts suggested that due to the specificity of the survey respondents, the scale needed to be trimmed and optimized to make the questionnaire concise and clear and more practical to administer. They were involved in the review and revision of the questionnaire, and ultimately the percentage of absolute agreement on the accuracy and brevity of the presentation of the revised questionnaire was 100%. In the third phase, a pilot test was conducted with 20 patients to assess the face validity of the questionnaire items, evaluate their logical consistency, judge comprehensibility, and improve their wording. These patients also made suggestions regarding the format and wording of the questions. Ultimately all patients found the questionnaire to be reflective of healthcare priorities and easy to complete. We incorporated these suggestions into the revised questionnaire. After three phases of content and validity testing, the revised questionnaire was translated into Chinese, and we used a back-translation method to ensure the consistency of the original Chinese and English versions of the questionnaire. Seven relevant experts who also participated in the previous phases of content and face validity testing checked the Chinese versions of the questionnaire, and ultimately, the percentage of absolute agreement on the accuracy of expression of the Chinese versions was 100%.

Table 2 shows the final version of the scale. Patient compliance consisted of three indicators that were adapted from the scale proposed by Verleye et al. [12] and Dagger et al. [44]. Patient loyalty was measured with two indicators adapted from Kim et al. [18], namely, return and referral, which were also considered important customer engagement behaviors by Kumar and Pansari [19]. Satisfaction was assessed with four indicators adapted from Verleye et al. [12], McKinnon et al. [45], and Dagger et al. [44]. Trust included three indicators from Alrubaiee and Alkaa’ida [46] and Chang et al. [22]. Responsiveness consisted of three indicators adapted from Mitropoulos et al. [25]. Empathy consisted of three indicators adapted from Dagger et al. [44] and Bendapudi et al. [47]. Surroundings included six indicators adapted from Sun et al. [48], Kim et al. [18], and Chang et al. [22]. Access consisted of four indicators from Kim et al. [18]. Most of the recent medical literature such as Verleye et al. [12], Kim et al. [18], and Chang et al. [22] used a five-point scale and patients who participated in the pretest indicated that a five-point scale was sufficient to accurately represent their attitudes. Thus, all the questionnaire items were assessed using a five-point Likert-type scale ranging from 1 to 5 (“strongly disagree” to “strongly agree”).

### 3.4. Analysis Strategies

This study employed the partial least square (PLS) technique of structural equation modeling (SEM) to estimate the proposed model. We chose PLS-SEM for two reasons. First, PLS is appropriate for nonnormally distributed data. This technique obtains robust results even if the data are highly skewed [49]. Second, PLS is suitable for analyzing complex models involving multiple variables [50]. Third, PLS can be used to perform analyses when the research is based on the integration of multiple theories [51]. We used a two-step approach that consisted of measurement model assessment and structural model assessment to test the proposed hypotheses.

## 4. Results

### 4.1. Measurement Model Assessment

Reliability was assessed using Cronbach’s alpha (α). As shown in Table 3, the α values of all the constructs were greater than the threshold value of 0.7, indicating that all constructs had good reliability. Convergent validity was assessed based on three criteria: loadings, average variance extracted (AVE), and composite reliability (CR). The thresholds for the loadings, AVE, and CR were 0.708, 0.5, and 0.7, respectively [50]. As shown in Table 3, all the convergent validity criteria were greater than the threshold values. Discriminant validity was evaluated using the Fornell–Larcker criterion and the heterotrait–monotrait ratio (HTMT) [52]. As indicated in Table 4, the square root values of the AVE of all the constructs were greater than their correlations with the other constructs. As shown in Table 5, all the HTMTs were lower than the threshold value of 0.9, indicating that all the constructs had good discriminant validity.

### 4.2. Structural Model Assessment

The structural model was assessed using two criteria: the coefficients of determination (R^2^) for the endogenous constructs and the sign and size of the path coefficients (β). The threshold for R^2^ was 0.2 [53]. The sign of the path coefficients should align with the associated hypothesis and the estimates should be statistically significant based on a 95% confidence interval (CI).

The R^2^ values of all the endogenous constructs were above the threshold, with 0.55 for satisfaction, 0.48 for trust, 0.37 for compliance, and 0.50 for loyalty; thus, our results had good explanatory power.

#### 4.2.1. Relationship between PEPMs and PEBs

As indicated in Table 6, patient satisfaction was significantly positively correlated with PEBs (compliance and loyalty) (β = 0.40, *p* < 0.001; β = 0.55, *p* < 0.001), thereby supporting H1a and H1b. Patient trust was significantly positively correlated with PEBs (compliance and loyalty) (β = 0.28, *p* < 0.001; β = 0.21, *p* < 0.001), thereby supporting H2a and H2b. Patient trust was significantly positively correlated with satisfaction (β = 0.22, *p* < 0.001), thereby supporting H3.

#### 4.2.2. Relationship between Functional Quality and PEPMs

Interaction quality dimensions (responsiveness and empathy) were positively associated with patient satisfaction (β = 0.32, *p* < 0.001; β = 0.21, *p* < 0.001), thereby supporting H4a and H4b. Surprisingly, the positive relationship between surroundings and satisfaction was not confirmed (β = −0.05, *p* > 0.1); thus, H4c was not supported. Except for interaction quality dimensions, access which is another functional quality dimension, was positively associated with satisfaction (β = 0.21, *p* < 0.001), thereby supporting H4d. Additionally, medical staff’s responsiveness and empathy were positively associated with patient trust (β = 0.25, *p* < 0.001; β = 0.51, *p* < 0.001), thereby supporting H5a and H5b.

#### 4.2.3. The Mechanism from Service Process to Behaviors

We further examined the mediating effect of patient satisfaction on the relationship between functional quality dimensions and PEBs. As reported in Table 7, patient satisfaction had a significant mediating effect between service providers’ responsiveness and PEBs (patient compliance and loyalty) (β = 0.13, *p* < 0.001; β = 0.17, *p* < 0.001). Patient satisfaction had a significant mediating effect between service providers’ empathy and PEBs (patient compliance and loyalty) (β = 0.09, *p* < 0.001; β = 0.12, *p* < 0.001). The indirect effects of surroundings on patient compliance and loyalty were not significant (β = −0.02, *p* > 0.05; β = −0.03, *p* > 0.05). Patient satisfaction had a significant mediating effect between access and PEBs (patient compliance and loyalty) (β = 0.08, *p* < 0.001; β = 0.11, *p* < 0.001). In addition, the 95% CIs of the indirect paths from responsiveness, empathy, and access to compliance and loyalty via satisfaction did not include 0, while the 95% CIs of the indirect paths from surroundings to patient compliance and loyalty via patient satisfaction did. Therefore, patient satisfaction served as a mediator between all the functional quality dimensions except surroundings and PEBs.

We also examined the mediating effects of trust on the relationship between interaction quality and PEBs. As reported in Table 7, patient trust had a significant mediating effect between service providers’ responsiveness and PEBs (patient compliance and loyalty) (β = 0.07 *p* < 0.001; β = 0.05, *p* < 0.001). Patient trust had a significant mediating effect between service providers’ empathy and PEBs (patient compliance and loyalty) (β = 0.14, *p* < 0.001; β = 0.11, *p* < 0.001). The 95% CIs of the indirect paths from responsiveness and empathy to compliance and loyalty did not include 0. Therefore, trust served as a mediator between interaction quality and PEBs.

## 5. Discussion

This paper sought to examine how functional quality encourages PEBs. Our results demonstrated that the responsiveness and empathy of service staff and access affect patient compliance and loyalty through patient satisfaction. The responsiveness and empathy of service staff affected PEBs through patient trust. These findings suggested the proposed model’s effectiveness for examining the antecedents of PEBs in medical services.

Our findings revealed that functional quality dimensions (responsiveness, empathy, and access) were positively associated with satisfaction. This finding agrees with the assertions of previous relevant studies. For example, Kim et al. [18] and Meesala and Paul [30] demonstrated that the empathy and responsiveness of contact employees had a positive impact on patient satisfaction. Berry et al. [34] emphasized the importance of access in a high-contact system and the need to schedule services based on customer need. However, the positive relationship between surroundings and satisfaction was not supported, unlike in previous studies [22]. This may be because medical services are a type of high-contact professional service, in which patients focus their attention on a series of interactions and ignore their surroundings. Furthermore, the hospitals we selected are equipped with high-quality facilities; thus, they already meet the needs of most patients. The role of surroundings can also be explained by Herzberg’s motivation–hygiene theory [54]: the good performance of surroundings can eliminate customer dissatisfaction but will not improve customer satisfaction. As such, our research provides a new direction for service process management in high-contact professional services.

Our findings revealed that positive interaction dimensions (service staff’s responsiveness and empathy) were positively associated with patient trust. This finding agrees with the assertions of uncertainty reduction theory, which suggests that customers gain trust to reduce their uncertainty by obtaining information directly from the target person [26].

Our findings revealed the mediating effect of patient satisfaction on the relationship between functional quality dimensions and PEBs. The positive indirect effect of responsiveness, empathy, and access on PEBs through patient satisfaction were supported in our findings, which extend the general path of service management from the patient engagement perspective [55,56]. Additionally, the positive indirect effects of responsiveness and empathy on PEBs through patient trust were supported in our findings. Related studies such as those on the trust–commitment model have highlighted the mediating effect of trust on the relationship between communication and behaviors such as compliance and cooperation [57]. Our findings confirmed the importance of patient trust identified by the trust–commitment model in the patient engagement field.

This study has important practical implications. First, our findings can help medical practitioners enhance their understanding of the antecedents of PEBs in medical services. Considering the characteristics of medical services (high-contact professional services), we call for more efforts directed toward improving service processes rather than simply relying on technical interventions. Specifically, hospitals and contact employees should devote time and effort to functional quality management to encourage PEBs.

Second, our findings reveal that hospitals should improve their functional quality in three dimensions, namely, responsiveness, empathy, and access. To ensure the empathy and responsiveness of service staff, professionals and other contact employees should ensure that they respond in a timely manner to any patient requests, express their willingness and ability to help patients, and motivate patients to actively express their needs and share personal information. Including patient-centered communication as part of the educational curriculum for medical students or providing decision aids to increase patient involvement as part of clinical guidelines are potential strategies for training physicians to become more compassionate and responsive. To ensure that patients’ needs are quickly met, hospitals should recruit sufficient front-line staff and properly manage the number of patients served by each individual. In addition, to provide patients with convenient access, more emphasis should be placed on optimizing appointment policies to accommodate patients’ appointment schedules to the greatest extent possible. Hospitals should timely and reliably communicate wait-time information, such as by providing waiting sequence numbers and estimated waiting times, to reduce patients’ perceived waiting times.

Third, our findings confirm the importance of patient trust and satisfaction as the PEPMs that drive PEBs. More effort should be made to cultivate patient trust and satisfaction. In addition to service process management, professionals bear the responsibility of gaining patient trust and satisfaction. Considering the high-contact nature of healthcare systems, administrators and healthcare providers should be dedicated to educating and training physicians to apply patient-centered communication to increase patient involvement and create a warm and pleasant communication atmosphere rather than simply providing patients with professional and technical data. Professional services should also be human.

Although this study has many valuable findings and implications, it remains preliminary and includes a number of limitations. First, this study collected data from the healthcare sector in China. Further research can explore the effectiveness of the model in other countries. Second, this study may not include all the important variables. Further research can consider other variables, such as organizational performance indicators, to provide managers with more practical implications. Third, this study used a survey as the main research method. Other methods, such as experiments, can be used to cross-validate the conclusions. Fourth, the questionnaires were completed by the same person during the survey process due to the purpose and nature of the study. Although we have done some ex ante control and ex post testing for common method variance, it cannot be eliminated completely. Further research can collect data from multiple sources. Finally, data for the paper were collected from two hospitals. While this study provides preliminary evidence on how to encourage PEBs from a functional quality perspective in high-contact professional services, the representativeness of the sample needs to be improved. Further research can collect data from multiple hospitals.

## 6. Conclusions

Considering the high-contact, professional nature of medical services, this study aims to explore how PEBs are encouraged from the perspective of functional quality. The results show that responsiveness, empathy, and access affect compliance and loyalty through satisfaction. The responsiveness and empathy of service staff affect PEBs through patient trust. This study thus enriches the literature on PEBs in the context of high-contact professional services.

## Figures and Tables

**Table 1:** Demographic characteristics.

Demographic Information		Frequency	Percentage (%)
Gender	Male	406	43.1
	Female	536	56.9
Age	<20	6	0.6
	20–29	192	20.4
	30–39	317	33.7
	40–49	199	21.1
	≥50	228	24.2
Occupation	Private sector	617	65.5
	Public service	122	13.0
	Student	73	7.7
	Other	130	13.8
Education	Junior high school or below	218	23.1
	Senior high school	198	21.0
	Bachelor’s degree	473	50.2
	Master’s degree and above	53	5.7

Note: “Other” refers to retired, unemployed, etc.

**Table 2:** Constructs and corresponding items.

Constructs	Items
Compliance	COM1. You will follow the doctor’s instructions [44]. COM2. You will adequately complete all expected behaviors [12]. COM3. You will perform all required tasks [12].
Loyalty	LOY1. You will choose this hospital the next time you need one [18]. LOY2. You will recommend this hospital to your family and friends [18].
Trust	TRU1. You believe that doctors in hospitals have adequate medical skills [46]. TRU2. You trust that all the hospital staff are kind and benevolent [46]. TRU3. You have confidence in the reliability and integrity of the medical services [22].
Satisfaction	SAT1. You are satisfied with the hospital’s services [12]. SAT2. You are satisfied with the treatment plan given by your doctor [44]. SAT3. You are satisfied with the attitude of the hospital staff [45]. SAT4. The hospital’s services meet your expectation [12].
Responsiveness	RES1. When you ask a question, you receive adequate answers or explanations from doctors and other healthcare providers [25]. RES2. The medical staff clearly explain the purpose, risks, and efficacy of tests/treatment when you ask [25]. RES3. The medical staff clearly explain what might happen during the test/treatment when you ask [25].
Empathy	EMP1. You have enough time to discuss your health issue with the doctor [47]. EMP2. The doctor is polite and listens carefully to you [44]. EMP3: You feel that you were treated like a VIP at the hospital [44].
Surroundings	SUR1. The hospital’s elevators are clean and spacious and are suitable at crowded times [48]. SUR2. The air ventilation, temperature, and conditions in the hospital were suitable [18]. SUR3. The toilet areas in the hospital were clean [22]. SUR4. The waiting area is very clean [18]. SUR5. The waiting area is comfortable and quiet [22]. SUR6. The lighting in the waiting area is comfortable [22].
Access	ACC1. The total time you spent waiting in the hospital was acceptable [18]. ACC2. Making appointments was convenient [18]. ACC3. Your appointment is on the day you wanted [18]. ACC4. Your appointment is at the time you wanted [18].

**Table 3:** Construct reliability and validity.

Constructs	Items	Loadings	α	CR	AVE
Responsiveness	RES1	0.81	0.76	0.85	0.66
	RES2	0.81			
	RES3	0.81			
Empathy	EMP1	0.81	0.72	0.84	0.64
	EMP2	0.87			
	EMP3	0.82			
Surroundings	SUR1	0.72	0.86	0.89	0.58
	SUR2	0.83			
	SUR3	0.80			
	SUR4	0.72			
	SUR5	0.71			
	SUR6	0.79			
Access	ACC1	0.80	0.89	0.92	0.75
	ACC2	0.86			
	ACC3	0.88			
	ACC4	0.81			
Satisfaction	SAT1	0.85	0.88	0.92	0.73
	SAT2	0.84			
	SAT3	0.82			
	SAT4	0.92			
Trust	TRU1	0.95	0.90	0.94	0.84
	TRU2	0.89			
	TRU3	0.91			
Compliance	COM1	0.88	0.85	0.91	0.76
	COM2	0.86			
	COM3	0.88			
Loyalty	LOY1	0.93	0.86	0.94	0.88
	LOY2	0.94			

Note: α, Cronbach’s alpha; CR, composite reliability; AVE, average variance extracted.

**Table 4:** Correlations between constructs.

Constructs	1	2	3	4	5	6	7	8
1. Responsiveness	**0.81**							
2. Empathy	0.62	**0.80**						
3. Surroundings	0.54	0.47	**0.76**					
4. Access	0.37	0.50	0.29	**0.86**				
5. Satisfaction	0.62	0.63	0.38	0.50	**0.86**			
6. Trust	0.56	0.66	0.47	0.40	0.60	**0.92**		
7. Compliance	0.44	0.45	0.39	0.31	0.57	0.52	**0.87**	
8. Loyalty	0.49	0.54	0.31	0.52	0.68	0.54	0.41	**0.94**

Note: The bold numbers in the diagonal row are square roots of the AVE.

**Table 5:** Heterotrait–monotrait ratio (HTMT).

Constructs	1	2	3	4	5	6	7	8
1. Responsiveness								
2. Empathy	0.81							
3. Surroundings	0.63	0.59						
4. Access	0.43	0.62	0.32					
5. Satisfaction	0.70	0.78	0.44	0.57				
6. Trust	0.65	0.82	0.53	0.45	0.67			
7. Compliance	0.51	0.58	0.46	0.36	0.66	0.59		
8. Loyalty	0.57	0.67	0.36	0.59	0.78	0.62	0.47	

**Table 6:** Results regarding direct effects.

Path	β	t-Values
Gender → Compliance	0.01	0.78 ^NS^
Gender → Loyalty	–0.06	2.56 **
Age → Compliance	–0.01	0.34 ^NS^
Age → Loyalty	–0.01	0.54 ^NS^
Education → Compliance	–0.01	0.33 ^NS^
Education → Loyalty	–0.00	0.06 ^NS^
Occupation → Compliance	–0.02	0.90 ^NS^
Occupation → Loyalty	0.04	1.79 ^NS^
Satisfaction → Compliance	0.40	10.27 ***
Satisfaction → Loyalty	0.55	13.13 ***
Trust → Compliance	0.28	7.94 ***
Trust → Loyalty	0.21	8.65 ***
Trust → Satisfaction	0.22	9.36 ***
Responsiveness → Satisfaction	0.32	10.62 ***
Empathy → Satisfaction	0.21	9.07 ***
Surroundings → Satisfaction	–0.05	1.37 ^NS^
Access → Satisfaction	0.21	7.28 ***
Responsiveness → Trust	0.25	4.84 ***
Empathy → Trust	0.51	10.86 ***

Note: Gender was coded 1 for females and 0 for males; education was coded 1 for junior high school or below, 2 for senior high school, 3 for a bachelor’s degree, and 4 for a master’s degree or above; Occupation was coded as 1 for the private sector and 0 for other. *** *p* = 0.001, ** *p* = 0.01, * *p* = 0.05, ^NS^ = not significant (based on a Student’s t (4999) distribution with two tails).

**Table 7:** Results regarding indirect effects.

Mediator	Path	β	t-Values	CI	
				2.5%	97.5%
Satisfaction (functional quality and PEBs)	Responsiveness → Satisfaction → Compliance	0.13	7.33 ***	0.10	0.17
	Responsiveness → Satisfaction → Loyalty	0.17	8.15 ***	0.13	0.21
	Empathy → Satisfaction → Compliance	0.09	6.06 ***	0.08	0.15
	Empathy → Satisfaction → Loyalty	0.12	7.41 ***	0.11	0.19
	Surroundings → Satisfaction → Compliance	–0.02	1.38 ^NS^	–0.03	0.01
	Surroundings → Satisfaction → Loyalty	–0.03	1.34 ^NS^	–0.04	0.01
	Access → Satisfaction → Compliance	0.08	6.01 ***	0.06	0.11
	Access → Satisfaction → Loyalty	0.11	5.93 ***	0.07	0.14
Trust (interaction quality and PEBs)	Responsiveness → Trust → Compliance	0.07	3.79 ***	0.02	0.07
	Responsiveness → Trust → Loyalty	0.05	4.34 ***	0.03	0.07
	Empathy → Trust → Compliance	0.14	6.46 ***	0.07	0.13
	Empathy → Trust →Loyalty	0.11	6.29 ***	0.08	0.14

Note: CI, confidence interval; *** *p* = 0.001, ** *p* = 0.01, * *p* = 0.05, ^NS^ = not significant (based on a Student’s t (4999) distribution with two tails).
